# Long bone shaft metastasis: a comparative study between cement filling and intercalary prosthesis

**DOI:** 10.1186/s12957-023-03242-z

**Published:** 2023-11-30

**Authors:** Yichao Fan, Boya Zhang, Liangyv Guo, Weitao Yao

**Affiliations:** Department of Bone and Soft Tissue Cancer, The Affiliated Cancer Hospital of Zhengzhou University & Henan Cancer Hospital, No. 127 Dongming Road, Zhengzhou, 450008 China

**Keywords:** Bone metastases, Palliative surgery, Internal fixation, Individual intercalary prosthesis, Clinical effect, Complications

## Abstract

**Background:**

Metastatic bone lesions in the extremities can cause severe pain and pathological fractures, significantly affecting patients’ quality of life. Timely intervention and effective management of long bone metastases can positively influence patient outcomes, including survival rates and subsequent treatment options.

**Objective:**

The objective of this study is to compare the efficacy and associated complications of two surgical reconstruction techniques and propose a more effective limb reconstruction approach for long bone metastases.

**Methods:**

A retrospective study was conducted on 28 patients with complete clinical data who underwent a surgical procedure for long bone metastases of the extremities in our department between January 2017 and June 2022. The patients were divided into two groups based on their surgical methods. In group 1, the affected bones were curetted and filled with cement, then secured with plates or intramedullary nails. In group 2, the affected bone segments were completely removed and replaced with custom intercalary prostheses. Various factors, including general patient information, surgical details, surgical effectiveness, and common complications, were compared and analyzed.

**Results:**

There were no significant differences in general patient information between the two groups, including age, gender, surgical site, and primary tumor type. The operative times were 115.37 min for group 1 and 108.90 min for group 2, respectively (*p* > 0.05). However, intraoperative blood loss differed significantly between the groups, with 769 ml in group 1 and 521 ml in group 2 (*p* < 0.05). The postoperative MSTS scores were 91% for group 1 and 92% for group 2 (*p* > 0.05). Postoperative complications included two cases of internal fixation failure and three cases of tumor recurrence in group 1, resulting in a 33% incidence rate, while group 2 experienced a 15% incidence rate with two cases of internal fixation failure.

**Conclusion:**

The results of this study suggest that both surgical techniques are effective for the treatment of long bone metastases of the extremities. However, the custom intercalary prostheses technique in group 2 showed a lower incidence of complications and less intraoperative blood loss. Therefore, it may be a more effective limb reconstruction approach for long bone metastases. Further studies with larger sample sizes are needed to confirm these findings.

**Supplementary Information:**

The online version contains supplementary material available at 10.1186/s12957-023-03242-z.

## Background

Bone metastases in the long bones of the extremities are a commonly observed phenomenon that primarily spreads through hematogenous spread [1, 2]. The epiphyses and metaphyses are the most frequently affected sites, followed by the diaphyses [3]. Since long bones play a crucial role in supporting movement and activity, the most significant complications associated with bone metastases include cancer-related pain and pathological fractures, with a 10–30% likelihood of the latter occurring [4]. Initial treatment for intramedullary lesions in long bone metastases primarily involves pharmacological prevention [5, 6], such as Denosumab or Zoledronic acid, and local radiotherapy [7, 8]. However, when cortical destruction exceeds one third of the circumference, the risk of pathological fracture significantly escalates, necessitating the reinforcement and repair of the affected bone. At this point, conservative treatment does not lead to significant bone healing. Specifically, the infiltration of tumors into the long tubular bones of the limbs can lead to cortical bone destruction and a subsequent loss of strength in the long bones. This degradation can significantly hinder daily activities like weight-bearing and walking. Therefore, many cancer surgeons prefer more aggressive treatments to provide lasting relief. More and more studies have shown that patients with metastatic fractures of long bones of extremities can steadily benefit from surgery [9–11].

Different surgical options have been described in previous studies, commonly employed methods include palliative surgeries, such as plate or intramedullary nail fixation, tumor scraping, and bone cement filling [9, 12–14]. These surgeries can immediately restore patient stability and significantly alleviate pain, making them a suitable approach for patients with shorter survival times (less than 3–6 months). However, these procedures often come with complications such as loosening and breakage of internal fixations, tumor recurrence, and restricted limb movement [4]. Prosthetic replacement is another commonly used approach for reconstructing malignant tumors in periarticular or metaphyseal regions [15]. Prostheses offer several advantages, including the complete resection of the tumor, strong enough, robust fixation, immediate postoperative stability, and long-term durability [16, 17]. However, when long bone metastases typically occur in the diaphysis or near the epiphysis, tumor replacement often requires the removal of normal epiphyseal bone, the affected joint, and its capsule, as well as surrounding tendons and ligaments. This could potentially compromise postoperative joint stability and range of motion.

As the range of treatment options for malignant tumors expands and patient survival time increases, the need for more effective methods of tumor removal and reconstruction to preserve normal activity during the limited survival period of patients has become a critical issue in the management of long bone metastases in the limbs. In recent years, the advent of digital technology and customized prostheses has made intercalary endoprostheses a more feasible and personalized option compared to traditional joint prostheses [10, 18]. The utilization of these prostheses eliminates the need for removing normal joint structures, thereby preserving the complete structure and function of the joint. Moreover, the tumor can be surgically removed en bloc, and the prosthesis can be precisely tailored to match the resected bone defect, ensuring optimal stability.

The objective of this study is to retrospectively analyze and compare the two reconstructive methods for bone metastases at our center: tumor scraping with bone cement filling and internal fixation reconstruction (group 1) and intercalary prosthesis reconstruction (group 2). We aim to assess the advantages and disadvantages of these methods, with the goal of identifying a more practical and safer approach for managing bone metastases in the extremities. The relevant data is presented below.

## Patients and methods

### Patient characteristics

We conducted a retrospective study to investigate patients with bone metastases in the extremities who received treatment at our department from January 2017 to June 2022. Out of the cases of long bone metastases, 56 were deemed eligible, and postoperative follow-up information was available for 28 patients (Supplementary Fig. 1 for details).

Among the 28 patients, 7 cases (25.00%) involved the humerus of the upper extremity, 15 (53.57%) were located in the femur, and 6 (21.43%) were found in the tibia of the lower extremity. All patients reported experiencing local pain and limited range of motion in the affected limb. Preoperative pathological fractures were observed in 11 cases (39.29%).

The primary disease was confirmed as lung cancer in 11 cases (39.29%), breast cancer in 5 cases (17.86%), kidney cancer in 4 cases (14.29%), thyroid cancer in 1 case (3.57%), and other malignant tumors in 7 cases (25.00%). Treatment for the primary disease was ongoing in 18 cases (64.29%), while metastases were initially diagnosed as long bone metastases or during follow-up of the primary disease in 10 cases (35.71%).

### The treatment process

#### Indications for surgery

All patients underwent a multidisciplinary team (MDT) consultation and preoperative assessment. Patients with an ECOG score of 0–2, an expected survival time longer than 3 months, a risk assessment for pathological fracture of the long limb (Mirels score [19, 20]) greater than 9, and generally normal blood count, liver, and kidney function, were considered suitable for surgical treatment.

#### Contraindications

Patients with deep vein thrombosis (including venous cancer thrombosis), challenging-to-correct electrolyte disorders, compromised vital organs such as heart, brain, or lung function, concurrent paraplegia, severe limb swelling with significant vascular nerve involvement, and difficulties in limb preservation were considered unsuitable candidates for the surgery.

#### Surgical procedure

The patient is placed in the supine position with the affected limb elevated. Routine towel disinfection was performed, and according to the location of the patient’s tumor, the appropriate surgical approach was selected and the tumor bone was exposed by blunt dissection.

##### Group I: Tumor bone scraping and internal fixation

In patients with an affected limb, the tumor bone is exposed through blunt dissection in the intermuscular space. To prevent intraoperative fractures, an initial step involves placing a plate distally and proximally to the long bone. The tumor bone is then carefully grooved, and the tumor tissue is meticulously scraped from both the inside and outside of the medullary cavity. At both ends of the fracture, a titanium intramedullary pin with a diameter of approximately 2 mm is inserted into the medullary cavity (to facilitate postoperative MRI). Subsequently, the bone defect area, as well as the distal and proximal medullary cavity, is meticulously filled with bone cement.

##### Group II: Prosthetic replacement

The proximal and distal osteotomy lines are determined based on the preoperative imaging data. The surrounding normal muscle is carefully dissected, followed by osteotomy, release, and complete excision of the diseased intercalary bone. The distal and proximal marrow cavities are then expanded, and an intercalary prosthesis is carefully fitted, ensuring the maintenance of the normal force line and anatomical position of the limb. Finally, the prosthesis fixation screws are inserted and securely tightened.

#### Post-operation and follow-up

Prophylactic anti-infection measures are initiated within 24–48 h of surgery. Postoperative rehabilitation of the affected limb is gradually started, and patients are able to walk independently at 3–6 weeks post-operation. Additional postoperative therapies for the primary cancer are initiated at 1 month postoperatively. Selective radiotherapy is administered to the surgical area where internal fixation is performed after surgery.

A regular follow-up of each patient was conducted until June 2022 or death. Patients are regularly followed up to monitor swelling, pain, and movement of the limb. Plain radiographs are taken to assess if the fixations have loosened and if the tumor has relapsed. If necessary, an MRI was to be performed to confirm tumor recurrence. Patients abide by the follow-up schedules: once every 3 months within 1 year after the surgery; 1–2 years after the surgery, once every 4 months; 2–3 years after the operation, once every 5 months; 3–5 years after the operation, twice a year; 5 years after the operation, once a year. Survival was defined as the time from surgery to the last follow-up or death.

#### Evaluation of limb function and common complications

All patient analysis is conducted with regard to survivorship, complications, site of complication, functional outcomes, and fixation method. The MSTS score [21] is utilized to assess limb function, while the range of motion of the adjacent joints is also recorded.

Postoperative complications may include superficial infection, deep interstitial infection, implant loosening and rupture, and tumor recurrence. The time of occurrence and consequences of treatment are also documented in detail.

#### Statistical analysis

Statistical outcomes were measured using the chi-square test and *t*-test for comparative studies for measurement data, with a level of significance set at *p* < 0.05, utilizing SPSS 20.0 software.

## Results

### General information

There were 15 male and 13 female patients included in the study, with an average age of 58.09 years. There were no statistically significant differences between the two groups regarding gender, age, site of surgery, primary tumor, and previous treatment. General information for both groups is presented in Table 1 and was not found to be statistically different (*p* > 0.05).

**Table 1:** General information of the two groups

General information		Group 1	Group 2	*P* value
Gender (cases *n*)	Male	8	6	0.62
	Female	7	7	
Age (years) (mean ± SD)		56.83 ± 7.64	62.08 ± 4.21	0.78
Locations (cases *n*)	Humerus	5	2	0.43
	Femur	7	8	
	Tibia	3	3	
Length of tumor (cm)		12.50 ± 2.51	13.06 ± 2.78	0.86
Pathological fracture (cases *n*)	Yes	6	5	0.64
	No	9	8	
Primary cancer (cases *n*)	Lung	6	5	0.78
	Breast	3	2	
	Renal	2	2	
	Thyroid	1	0	
	Others	3	4	
Therapeutic process (cases *n*)	Yes	10	8	0.71
	No	5	5	
Survival times (months) (mean ± SD)		17.07 ± 7.73	21.30 ± 8.17	0.28

*Group 1* tumor curettage and fixation, *Group 2* intercalary stem prosthesis reconstruction

### Operative information

The operative time for group I was 115.37 min, while for group II, it was 108.90 min (*p* > 0.05). The intraoperative bleeding was 769 ml for group I and 521 ml for group II, with a statistically significant difference (*p* < 0.05). However, the MSTS scores for both groups were 91% and 92%, respectively, with no statistically significant difference (*p* > 0.05).

### Postoperative follow-up and complications

Patients were regularly followed up after surgery. The function of the affected limb, with the support of a brace, generally returned to normal within 1–3 months post-surgery. Patients were able to resume normal daily activities without experiencing local discomfort, such as pain, at 3 months after surgery (Table 2).

**Table 2:** Clinical results of the two groups

Operational information		Group 1	Group 2	*P* value
Operation last time (minutes, mean ± SD)		115.37 ± 32.66	108.90 ± 27.51	0.19
Blood lose (ml, mean ± SD)		769.33 ± 206.82	520.92 ± 177.50	0.03^*^
MSTS score (%))		90.63 ± 5.77	92.05 ± 5.41	0.72
Complication rate (%))		33.33	15.38	0.01^*^
Complication type (cases *n*)	Hardware failure	2	2	
	Tumor relapse	3	0	

*Group 1* tumor curettage and fixation, *Group 2* intercalary stem prosthesis reconstruction

^*^Indicated *p* < 0.05

In terms of postoperative complications, group 1 had 2 cases of internal fixation failure and 3 cases of tumor recurrence, resulting in an incidence rate of 33%. In group 2, there were 2 cases of internal fixation failure, with an incidence rate of 15% (*p* < 0.05). Internal fixation failure occurred between 2 and 5 years after surgery, while tumor recurrence occurred between 1 and 3 years after surgery. Fortunately, there were no reported cases of wound infections or hematomas.

**Fig. 1:**
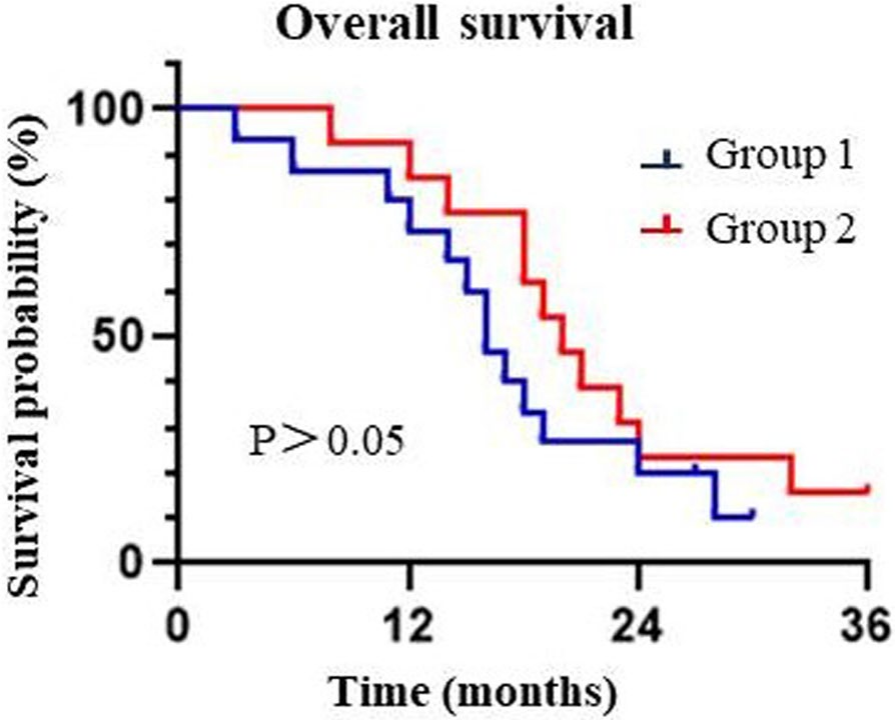
Kaplan‑Meier survival curves of the two groups according to the mode of operation (*p* > 0.05). In group 1, the involved bones were scraped and filled with cement and fixed with plates or intramedullary nails. In group 2, the involved bones were completely resected and replaced with individually intercalary prostheses

The survival time of patients in group 1 ranged from 3 to 30 months, with an average of 17.1 months. In group 2, the survival time ranged from 8 to 36 months, with an average of 21.3 months. The 12-month survival rates for the two groups were 73.3% and 84.6%, respectively (*p* > 0.05) (Fig. 1).

## Discussion

The intercalary prosthesis offers several advantages in the treatment of bone metastases. It is easy to use, provides firm fixation, and allows for a simple surgical procedure. Additionally, it enables complete removal of the local bone tumor, reducing the risk of recurrence after surgery [9–11, 22]. This study reports the results of cement filing and intercalary prosthesis treatment in patients with long-bone diaphyseal metastases of the extremities. The purpose of this study is to compare the efficacy and related complications of the two surgical reconstruction techniques, optimize the treatment strategy, and propose a more effective limb reconstruction approach for long bone metastases. The findings of this study support our hypothesis that intercalary prosthesis shows lower postoperative complication rates, shorter operative time, and less intraoperative bleeding compared with cement filling. Therefore, intercalary prosthesis may be a more effective limb reconstruction approach for long bone metastases.

Skeletal-related adverse events (SREs), including pathological fractures, pain, and hypercalcemia, are frequent complications in metastatic bone cancer [23–25]. These complications substantially affect the patient’s quality of life [24] and can lead to severe outcomes, such as deep vein thrombosis, pneumonia, paraplegia, and even death [26]. As such, early identification, prevention, and management of SREs are critical in the treatment of metastatic bone cancer [6, 23]. Pain in bone metastases is often a result of local tumor growth, irritation, or compression of the periosteum and nerves within the bone marrow. This pain can be effectively managed with oral analgesics and localized radiotherapy [27]. Pathological fractures occur when tumors cause destruction of the cortical and trabecular bone, leading to weakened bone strength and subsequent fracture. It is important to emphasize that the treatment of patients with bone metastasis requires a collaborative, multidisciplinary approach that focuses on pain relief and bone repair, while also considering individualized treatment plans [28, 29].

The primary goal of surgical intervention for metastatic long bone fractures in the extremities is to provide immediate reinforcement of the fracture and alleviate pain. Currently, palliative procedures such as internal fixation using intramedullary nails or plates are commonly employed for the treatment of pathological fractures in long bones [30]. This approach aims to restore the biomechanical integrity of the affected bone, providing immediate reinforcement of the fracture and alleviating pain. However, it is important to note that due to the incomplete removal of the local tumor, there is a risk of postoperative tumor recurrence and failure of the internal fixation. This method is primarily suitable for frail patients with heavy tumor burden and a limited life expectancy. On the other hand, metastatic patients with slower tumor progression or better tumor control often have a longer survival time and therefore require more durable internal fixation and complete removal of the metastatic cancer lesions. In such cases, the use of tumor prostheses and joint replacements is considered a more appropriate surgical option. Conventional modular tumor prostheses can be used for metastases located near the joint [31, 32]. However, in cases of diaphyseal metastases, joint replacement often involves the removal of a significant amount of healthy bone, resulting in the loss of muscle and ligament attachment points. This can lead to a loss of strength in the affected limb after surgery. Moreover, the use of prostheses in diaphyseal metastases carries a higher risk of postoperative loosening or breakage, making it a less ideal method for such cases.

The intercalary prosthesis is a relatively new type of prosthesis that has been developed for the treatment of long bone tumors [33]. It consists of an intramedullary pin at each end and a prosthetic part in the middle. There are two types of intercalary prostheses, namely the lap-tap connection and the male–female conical connection. The intercalary prosthesis is an individually designed product, and precise preoperative planning is required to ensure optimal outcomes. This involves inputting tumor-related imaging data, such as CT and MRI, into a computer and fusing them into three-dimensional digital graphics. This allows for accurate outlining of the extent of the tumor to be removed. The length and diameter of the reconstructed prosthesis, as well as the extent of fixation of the distal and proximal intramedullary stems involving bone, should be accurately designed. To reduce the risk of prosthesis loosening caused by the large metaphyseal medullary cavity and shorter stem, a larger diameter of the intramedullary stem and the addition of a flanking plate are used. The flanking plate helps distribute some of the load on the prosthesis, and the riveting of the locking holes in the stem and the locking screws in the flanking plate further enhances the stability of the stem in the medullary cavity. During the fitting process, it is crucial to ensure normal lines of force and angles of rotation of the limb to avoid early postoperative loosening or stress concentration fractures. At our center, we commonly use the lap-tap joint type of prosthesis because it does not require excessive stretching of the limb for repositioning. During the surgery, the prosthesis is pre-fitted to determine its basic position and identify the screw holes for the lateral plates. Once the prosthesis stem is fixed with bone cement, the flanking plate is fitted, and the screws of the connector are secured.

**Fig. 2:**
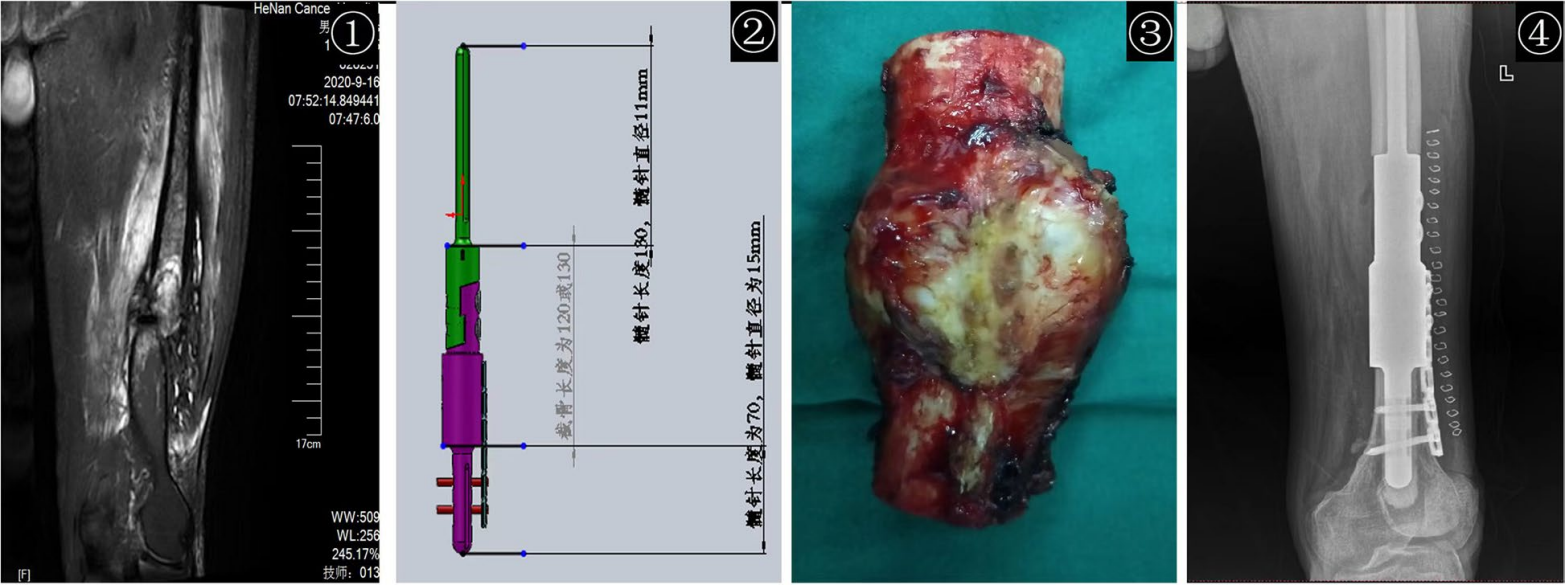
En bloc resection and intercalary prosthesis implantation for the treatment of left femoral shaft bone metastasis, male, 60 years old. ① Preoperative MRIT2WI (coronal plane) showed that the left distal femur showed mass-like long T2 signal, indicating tumor signal and pathological fracture, which was consistent with the diagnosis of osteolytic bone metastasis. ② The design diagram of customized Intercalary prosthesis. ③ Bone segment with en bloc resection. ④ The positive X-ray films were reexamined 24 months after the implantation of the intercalary prosthesis

**Fig. 3:**
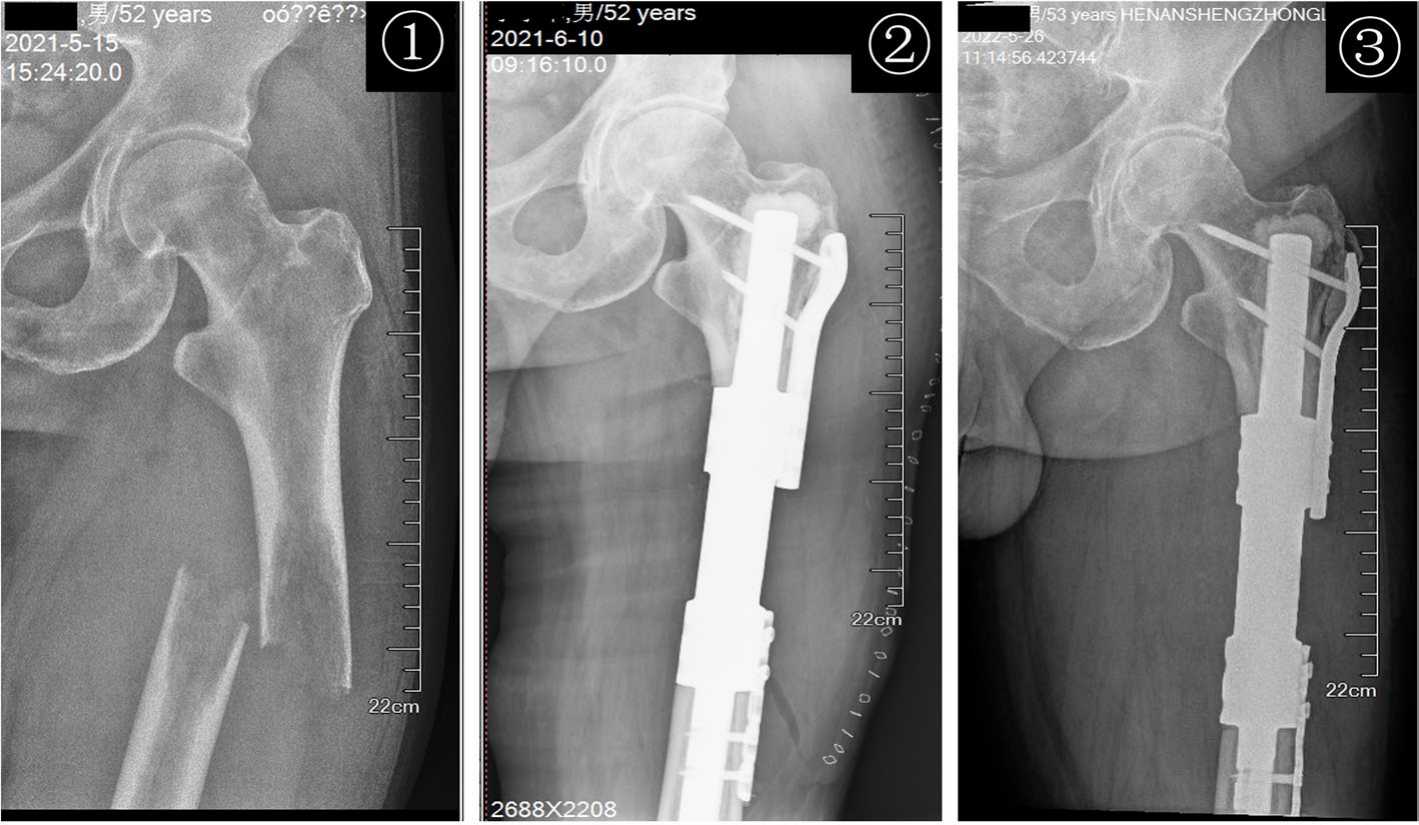
Imaging follow-up process of segmental intercalary prosthesis. ① Positive X-ray of the left femur showing pathological fracture of the shaft due to metastatic tumor, there were osteolytic destruction, discontinuity of bone cortex, obvious fracture line, and angular deformity at the proximal end of the left femoral shaft. ②–③ One month and 12 months after the operation, the positive X-ray films of the proximal femur showed that the prosthesis was in a good position

**Fig. 4:**
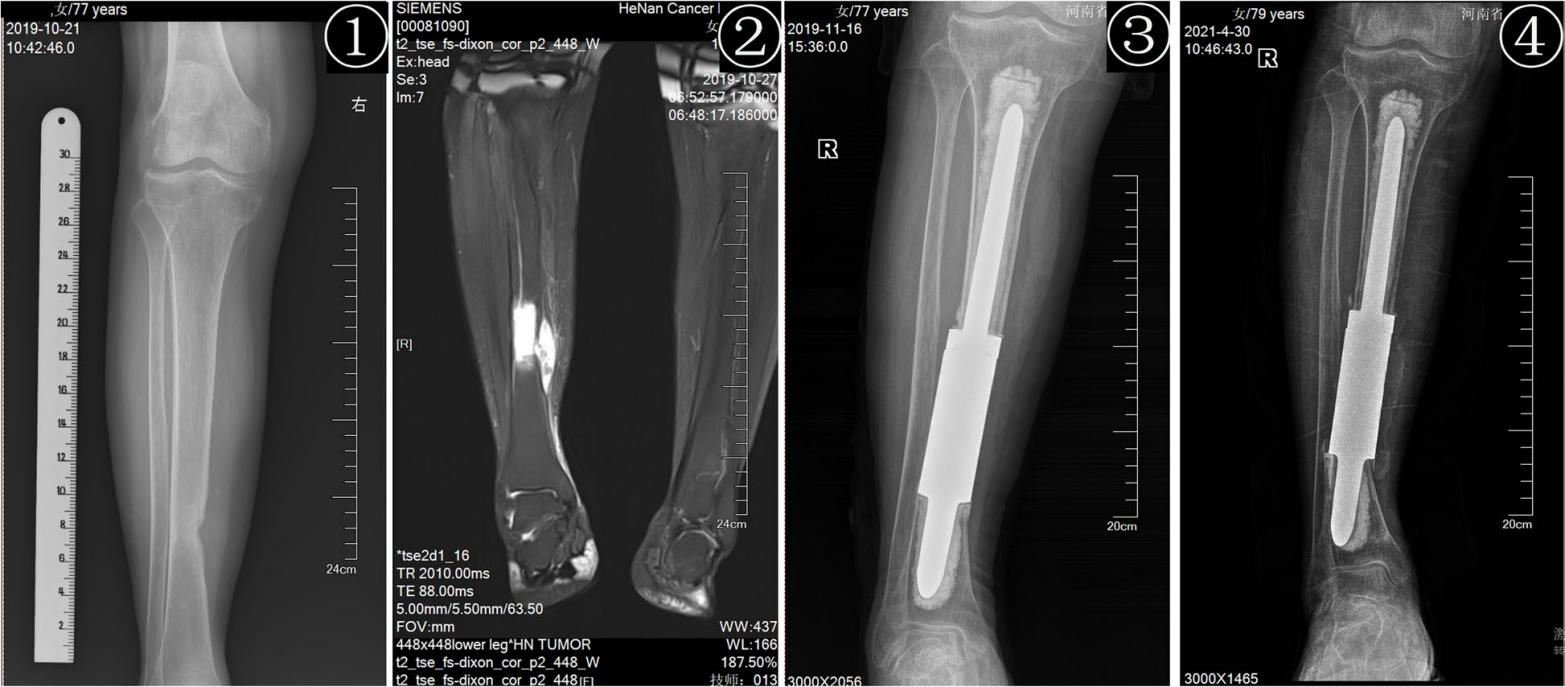
Metastatic tumor of the right distal tibia, female, 79 years old. ① the preoperative anteroposterior radiographs showed osteolytic destruction of the tibial shaft. ② Preoperative MRI T2WI (coronal) showed that the right middle tibia showed a mass-like long T2 signal, indicating the tumor signal. ③–④ The positive X-ray films of the tibial diaphysial prosthesis at 1 month and months after the operation

**Fig. 5:**
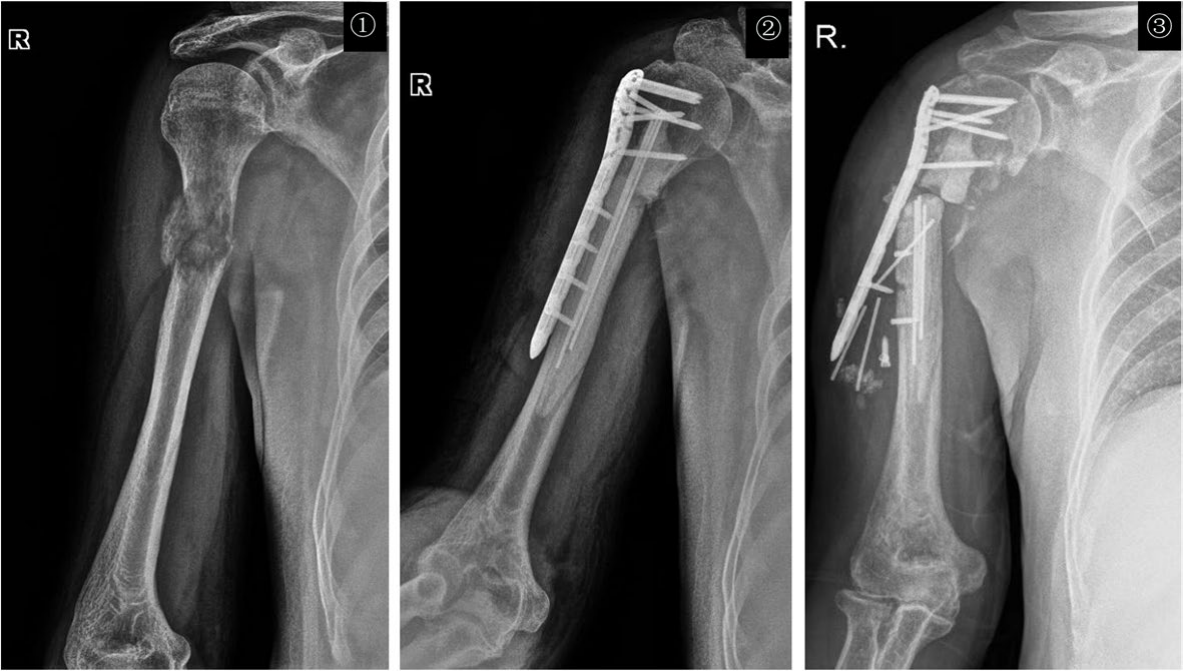
The metastatic tumor of the proximal humerus was treated with curettage and cement fixation, female, 61 years old. ① A positive X-ray of the right humerus showed a pathological fracture of the diaphysis of the humerus due to a metastatic tumor. ② Anteroposterior radiographs of the proximal humerus one month after surgery. ③ Anteroposterior radiographs of the proximal humerus half years after the operation showed a fracture of the proximal humerus and the hardware failure

**Fig. 6:**
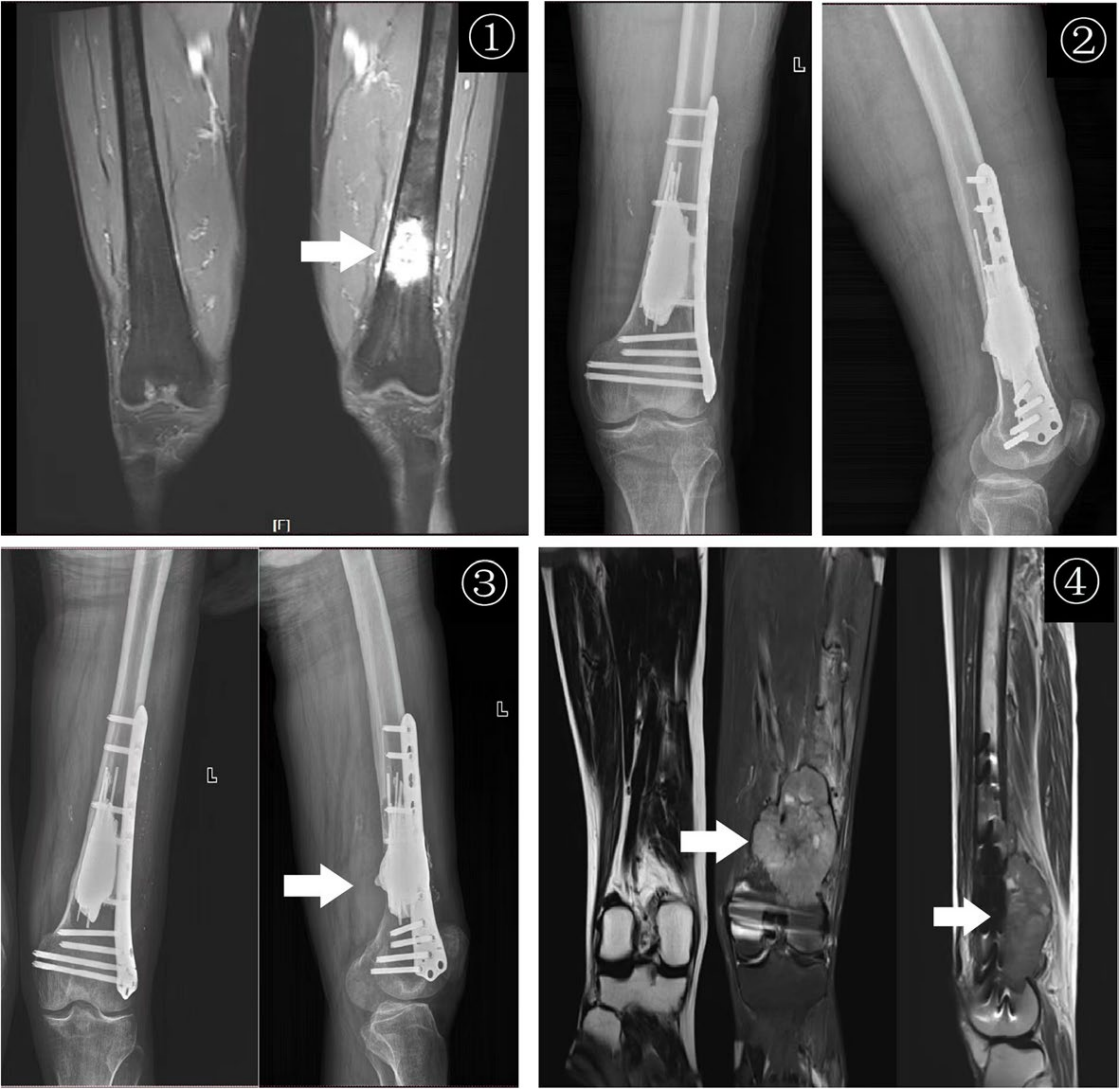
Metastatic tumor of the distal femur of renal cell carcinoma, male, 53 years old. ① Preoperative MRI T2WI (coronal) showed that the left distal femur showed a mass-like long T2 signal, indicating a tumor signal. ②–③ After 2 and 6 months of curettage and cement fixation of the tumor, the X-ray films (positive and lateral position) showed that the cement-host bone healed completely. ④ Ten months after the operation, MRI showed that the mass (coronal and sagittal) was slightly longer on T1 and T2 signal intensity, indicating tumor recurrence

In our study, we found that intercalary prosthesis replacement surgery had a mean operative time of 108.90 ± 27.51 min and an intraoperative blood loss of 520.92 ± 177.50 ml. Compared to the traditional surgical approach of tumor scraping with internal fixation, we have found that intercalary prosthesis replacement offers the advantages of less surgical bleeding and shorter operation times. Consistent with previous studies, involving a wide exposure, curettage and cement filing may require more operation time, blood loss, and recovery time than extensive resection and prosthesis reconstruction [9, 10, 22]. Additionally, functional training of the limb can begin shortly after surgery, and normal movement of the limb can be resumed within three weeks. The positioning of the customized prosthesis was satisfactory during the follow-up period, as shown in Figs. 2, 3, and 4. Furthermore, complete local tumor resection with the intercalary prosthesis eliminates the need for additional adjuvant therapies such as postoperative radiotherapy and reduces the risk of tumor recurrence after surgery. These findings are consistent with previous studies [22, 34]. However, it is important to note that there are potential complications associated with prosthesis implantation, such as loosening or breakage [11, 35, 36], as well as tumor recurrence. In our study, we observed no other complications during the follow-up period in the intercalary prosthesis group (group 2), except for two patients who experienced sterile prosthesis loosening. In contrast, in the traditional surgery group (group 1), 33% of patients experienced complications of tumor recurrence within their limited life expectancy, as shown in Figs. 5 and 6. Ruggieri and colleagues [35] studied the application of intercalary prosthesis in the treatment of metastatic humeral lesions, with a mean follow-up of 24.9 months and only 1 case of mechanical loosening at 30 months. Similar to the results of this study, Dengxing Lun et al. reported that the incidence of complications in the IP group was only 12.5%, compared with 68.8% in the Segmental Allograft (SA) group [11]. Therefore, the low incidence of postoperative complications in group 2 (IP group) makes intercalary prostheses a reasonable choice for patients with long bone metastases of extremities.

To reduce the recurrence rate of tumor curettage, adequate preoperative planning is essential for successful surgery. It is important to preoperatively plan for complete resection of extra-tumor metastases with a normal calf. In order to ensure intraoperative osteotomy lines, a disposable osteotomy guide, such as patient-specific instrumentation (PSI) [37], can be designed and printed. Alternatively, the osteotomy line can be determined intraoperatively based on anatomical landmarks. During the surgery, the level of the osteotomy line is introduced using PSI or anatomical landmarks. In cases where patients have developed a pathological fracture and shortening, the length of the tumor prosthesis must be determined based on the contralateral normal limb. Our research team believes that surgery is best performed 2 weeks after the fracture, when the hematoma has organized and the surrounding periosteum has formed. This increases the likelihood of complete removal of the lesion. For metastases that bleed heavily after fracture and are difficult to remove completely intraoperatively, postoperative local radiotherapy can be added, along with aggressive treatment of the primary disease, to reduce recurrence after surgery.

As with any new technique, prosthetic insertional reconstruction of long bone metastases in the extremities has not been standardized, and the available literature is sparse and disorganized [38]. However, there have been some studies evaluating the functional outcomes of patients who underwent intercalary prosthesis implantation. One study by Feifei Pu et al. [22] retrospectively analyzed the functional status of 21 patients with shaft metastases after intercalary prosthesis implantation. The average follow-up period was 22 months, and the functional status of the patients recovered to 90–93% of their preoperative levels. Another comparative study by Dengxing Lun and colleagues compared segmental allotransplantation (SA) and prosthesis implantation (IP) in the treatment of metastatic tumors of the femoral shaft complicated with pathological fractures. The MSTS score in the IP group was significantly higher than that in the SA group (IP, 26.7 ± 1.6 vs SA, 20.3 ± 1.5; *P* < 0.05) 1 month after the operation. However, there was no significant difference in the last follow-up [11]. Similarly, McGrath et al. reported a mean MSTS score of 23 (ranging from 18 to 27) after prosthesis implantation [39]. In our study, patients were able to return to normal daily activities 3 months after surgery without experiencing local pain or other discomforts. Moreover, the functional status of both groups had recovered by more than 90%, with group 1 recovering by 90.63 ± 5.77% and group 2 recovering by 92.05 ± 5.41%. There was no statistical significance in the last follow-up (*P* = 0.72). According to previous literature reported, the mean survival time of patients with long bone metastases of extremities implanted with prostheses was 9–19.46 months [11, 22, 34, 40, 41]. In this study, the average survival time of the two groups was 17.1 and 21.3 months, respectively, and the 1-year survival rate was 73.3% and 84.6%, respectively (*P* > 0.05). The differences in primary tumor, tumor location, and selection criteria may be the reasons for the different survival rates in different studies. In our study, we included some tumor types with a good prognosis (breast cancer, kidney cancer, thyroid cancer, etc.).

However, there are several limitations to our study. Firstly, it was a retrospective study conducted at a single center with a small sample size. To further confirm the difference in prognosis between the two groups, larger multicenter studies are needed. Secondly, the limited life expectancy of most patients due to the primary tumor made it difficult to obtain long-term follow-up results. This may have also affected the interpretation of functional outcomes. Lastly, the rarity of the indications and the possibility of patients opting out of surgery due to financial constraints and other factors may introduce various biases into our study. Further research is needed to address these limitations and provide more comprehensive and reliable evidence for the safety and effectiveness of intercalary prosthesis implantation in the treatment of long bone metastases.

## Conclusions

The results of this study suggest that both surgical techniques are effective for the treatment of long bone metastases of the extremities. However, the custom intercalary prostheses technique in group 2 showed a lower incidence of complications and less intraoperative blood loss. Therefore, it may be a more effective limb reconstruction approach for long bone metastases. Further studies with larger sample sizes are needed to confirm these findings.

### Supplementary Information

**Additional file 1.**

## Data Availability

The datasets used and/or analyzed during the current study are available from the corresponding authors on reasonable request.

## Abbreviations

SREs: Skeletal-related adverse events
MDT: Multidisciplinary team
PSI: Patient-specific instrumentation
SA: Segmental allotransplantation
IP: Prosthesis implantation
