# Supplementary figures and images for: Long bone shaft metastasis: a comparative study between cement filling and intercalary prosthesis
Source: World J Surg Oncol. 2023 Nov 30;21:374. doi: 10.1186/s12957-023-03242-z (PMC10687828; doi:10.1186/s12957-023-03242-z)

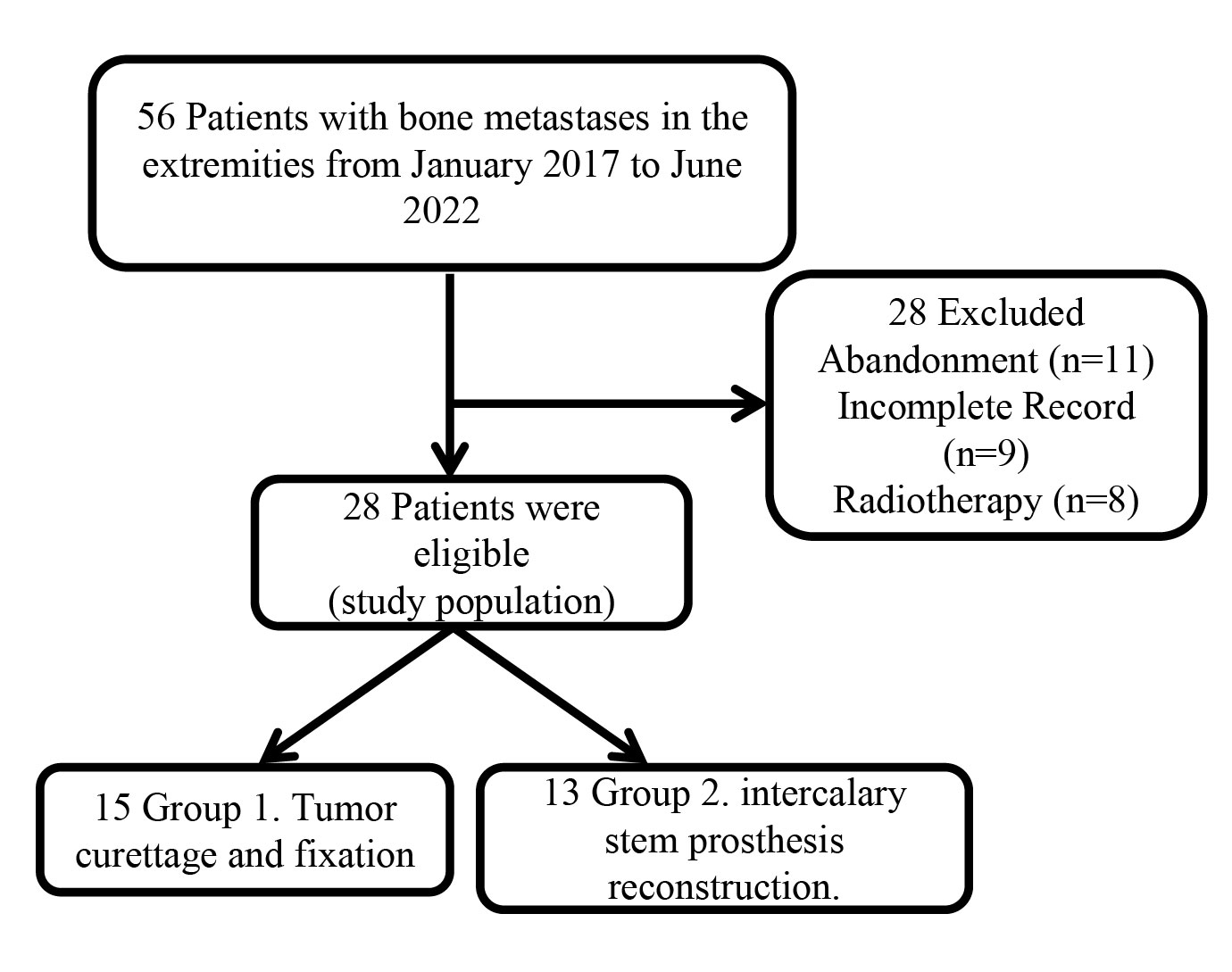

Supplement: Supplementary file 1 — Additional file 1. [file 12957_2023_3242_MOESM1_ESM.jpg]
